# Performance Evaluation of the Maxwell 16 System for Extraction of Influenza Virus RNA from Diverse Samples

**DOI:** 10.1371/journal.pone.0048094

**Published:** 2012-10-29

**Authors:** Hongbo Liu, Yan Gan, Bo Yang, Hui Weng, Chunmei Huang, Daofeng Yang, Ping Lei, Guanxin Shen

**Affiliations:** 1 Department of Immunology, Tongji Medical College, Huazhong University of Science and Technology, Wuhan, People’s Republic of China; 2 Virology Laboratory, Zhongshan Center for Disease Control and Prevention, Zhongshan, People’s Republic of China; 3 Neuroimmunology Laboratory, Barrow Neurological Institute, St. Joseph’s Hospital and Medical Center, Phoenix, Arizona, United States of America; 4 Department of Intensive Care Unit, Boai Hospital of Zhongshan, Zhongshan, People’s Republic of China; 5 Department of Infectious Diseases, Tongji Hospital, Huazhong University of Science and Technology, Wuhan, People’s Republic of China; Centers for Disease Control and Prevention, United States of America

## Abstract

This study evaluated the performance of the Maxwell 16 System (Promega) for extraction of influenza virus (flu-v) RNA from diverse samples compared to a classical manual method (QIAamp Kit, QIAGEN). Following extraction by the two methods, all samples were analyzed by Real-time RT-PCR. Results revealed that the use of the standard Maxwell 16 protocol (Maxwell 16-S) resulted in good linearity and precision across a wide concentration range and higher sensitivity of detection from flu-v stock suspensions than the manual method. Compared with the latter method, Maxwell 16-S extracted RNA more efficiently (higher RNA yield and/or fewer PCR inhibitors) from throat swabs and bronchoalveolar lavage fluids, while both methods performed comparably on fecal samples from human and poultry in terms of overall threshold cycle values and detection rates although the Maxwell 16-S co-purified more inhibitors from fecal samples. The capacity of this system to remove inhibitors from fecal matrix was improved by using a modified Maxwell 16 protocol with a reduced sample input, which eliminated all false-negatives produced by the Maxwell 16-S. These findings suggest that the Maxwell 16 System is suitable for RNA extraction from multiple-source samples for diagnosis of influenza and viral load determination and that a proper reduction in starting sample volume may improve the detection of flu-v from complex matrices such as feces. Additionally, this system allows flexible sample throughput and labor-saving sample processing with little or no risk of cross-contamination.

## Introduction

The grave threat posed either by the highly pathogenic avian influenza virus or by another emerging virus like the 2009 pandemic H1N1 (pH1N1) requires rapid laboratory detection of the first cases or clusters of influenza infection [1]. PCR-based nucleic acid (NA) assays are the first-choice techniques for flu surveillance and diagnosis due to their rapidity, sensitivity and specificity, although virus isolation is still critical for antigenic analysis and characterization of influenza virus (flu-v) [1], [2].

NA extraction is a crucial prerequisite for PCR analysis [3]. Conventional manual extraction methods are labor-intensive, susceptible to contamination and handling variations [4], [5]. The demand for automated systems has grown markedly as a result of increasing PCR testing since the onset of pH1N1 [2], especially at regional clinical and public health laboratories where specially trained staff are limited. Most automated extractors are designed to batch a significant number of samples and are not suitable for smaller laboratories because the costs of equipment, its maintenance, space requirements and need for disposables are prohibitive [5], [6]. Recently, the Maxwell 16 System (Promega, US), a compact and simple desk-top unit, was developed to extract viral total NA automatically from human plasma or serum samples based on a magnetic bead separation technique [7]. Its performance has been evaluated by the manufacturer using hepatitis B and C virus, cytomegalovirus samples, etc [7]. However, no formal assessment of extracting flu-v RNA via this system has so far been reported.

Therefore, in this study, we evaluated the performance of the Maxwell 16 System in extracting flu-v RNA for diagnosis of flu by using Real-time reverse transcriptase PCR (RRT-PCR). Several sample pretreatment procedures of this system were first investigated for the recovery of flu-v RNA. The system’s analytical sensitivity, linearity, precision and performance on clinical and field samples, reagent costs and extraction times were next compared with those of a commonly used column-based method (QIAamp Viral RNA Mini Kit; QIAGEN, Germany). The risk of cross-contamination during automated processing was also assessed.

## Materials and Methods

### Ethics Statement

This study protocol was reviewed and approved by the Ethics Committee of Huazhong University of Science and Technology (permit number S240). Written informed consent was obtained from all human participants on or before each study.

### Virus Stock

To ensure that all the samples could be handled safely within containment level 2 facilities, a pH1N1 isolate A/Zhongshan/SWL02/2009(H1N1) (pH1N1SWL02) was used as a representative human/avian flu-v for investigation of sample pretreatment, mock-infected sample preparation and evaluation assays. This virus was propagated and titrated in Madin-Darby canine kidney cells to contain 10^5.7^ 50% tissue culture infective doses (TCID_50_) per ml. After serial 10-fold dilution of the virus culture supernatant with viral transport medium (VTM), aliquots of the virus were stored at –70°C prior to use to avoid multiple freeze-thaw cycles.

### Clinical and Field Samples

#### Throat swabs

A total of 49 throat swabs were retrospectively selected for this study. These samples had been characterized by PCR as positive (strong, medium or weak) for pH1N1, seasonal flu A/H1, H3, or flu B virus.

#### Bronchoalveolar lavage fluids (BALFs)

32 BALFs were taken from patients in intensive care units with various diseases requiring bronchoalveolar lavage for diagnosis or treatment. After vortex for 2 min and centrifugation at 3000 rpm for 10 min, the supernatants of BALFs were tested by RRT-PCR with the CDC protocol [8]. One BALF was determined to be pH1N1 positive. The remaining 31 negative BALF supernatants without viscous phlegm were used to prepare mock-infected BALFs by mixing 100 µl of the pH1N1SWL02 stock (10^–4^ dilution) with 900 µl of the BALF supernatant.

#### Pooled fecal samples

Human feces were obtained from healthy subjects and fresh poultry droppings were collected from cages of live poultry in four markets. To increase heterogeneity of small samples, every three fecal samples from the same type of subject were combined into one mixture. Specifically, three 100-mg feces from three individuals were added to 3 ml VTM and homogenized by shaking for 20 min. The fecal suspensions (10%, W/V) were then centrifuged at 13000 rpm for 5 min, and the obtained supernatants were used as fecal matrices. The resulting 60 samples comprised chicken, duck, silkie, quail, pigeon, chukar (n = 7 for each) and human (n = 18) fecal pools. Ten poultry fecal pools were identified to be positive for flu A virus by a commercial RRT-PCR kit; 50 flu-v RRT-PCR negative fecal pools were used to prepare mock-infected samples by mixing 100 µl of the pH1N1SWL02 stock (10^–4^ dilution) with 900 µl of the fecal supernatant.

### RNA Extraction

#### Maxwell 16 system

An optimal temperature (56°C) and optimal time (10 min) were chosen and used for sample lysis in flu-v RNA extraction by this system in terms of RNA recovery from the pH1N1SWL02 stocks at high, medium and low concentrations (data not shown). After an initial lysis of sample followed by transfer of the sample lysate to Maxwell 16 LEV Cartridge, the remaining purification process was fully automated by the extractor in Viral Mode. The sample input of 200 µl and output of 50 µl, which were designated as standard Maxwell 16 protocol (Maxwell 16-S), were chosen based on the manufacturer’s recommendation and our pilot study. A modified protocol (Maxwell 16-M), in which both sample input and output were 100 µl, was also employed for flu-v RNA extraction from fecal samples.

#### QIAamp kit

RNA extraction using the QIAamp Kit was performed in parallel with the Maxwell 16 System for comparative evaluation. Purified RNA from 140-µl sample was eluted in 60-µl Buffer AVE (QIAGEN, Germany) according to the manufacturer’s instructions.

### RRT-PCR Assays

The CDC Flu A/B and RP primer/probe sets [8], [9] in combination with the CDC RRT-PCR protocol [8] were applied to the detection of universal flu A and B viruses, and human RNase P gene (RP) that serves as an internal positive control for human RNA. All samples were tested in duplicate on an ABI 7500 Fast System with the Superscript III Platinum One-step qRT-PCR Kit (Invitrogen, US). ROX reference dye was added at a final concentration of 50 nM to normalize the fluorescent reporter signal. The threshold cycle (Ct), representing the point at which amplification of NA is detected above background fluorescence, was used as a measure of relative RNA yield. The cut-off of the assays was set at a Ct-value of 40.

### Statistical Analysis

All statistical data were processed using SPSS 13.0 software. Linearity was assessed by regression analysis. The standard deviation for determination of inter- and intra-run coefficient of variation (CV) was calculated from one-way ANOVA. The Wilcoxon signed rank test was performed to compare Ct value differences between the two methods except that the paired *t*-test was used to compare Ct values of RP. The McNemar test was used for comparison of detection rates between the two methods. *P*<0.05 was considered as significant.

## Results

### Analytical Sensitivity and Linearity

**Figure 1:**
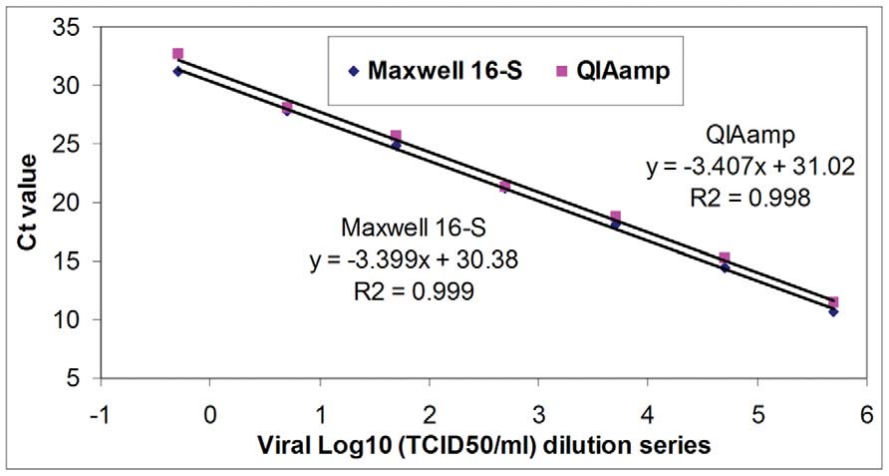
Linearity of both extraction methods in conjunction with Real-time RT-PCR assay. A 10-fold dilution series of influenza virus stock (10^5.7^ TCID_50_)/ml were extracted in triplicate for each dilution with the Maxwell 16-S and QIAamp Kit and then tested by the CDC Flu A Real-time RT-PCR assay. The virus concentration (log_10_TCID_50_/ml) was plotted against the mean Ct values of triplicate determinations for each dilution producing repeated positive results.

Dilution series ranging from pure to 10^–8^ of the pH1N1SWL02 stock were extracted in triplicate for each dilution by Maxwell 16-S and QIAamp Kit, respectively. The viral RNA was then tested by the CDC Flu A RRT-PCR. Good linearities were observed over a 7-log concentration range with R^2^-values of 0.999 and 0.998, and slopes of 3.399 and 3.407, respectively, for the Maxwell 16-S and QIAamp methods (Fig.1). Extraction by the automated procedure yielded lower Ct values (0.71 cycles on average) at all dilutions within the linearity range compared to the manual procedure. According to preliminary evaluation of sensitivity from data for the linearity experiment, dilutions of 10^–6^, 10^–7^ and 10^–8^ were chosen, and an additional seven replicates of the each dilution were extracted by Maxwell 16-S and QIAamp Kit for determination of analytical sensitivity. The results of 20 measurements in sum by RRT-PCR for each of the three dilutions are summarized in Table 1. After extraction with Maxwell 16-S, flu-v was detected in all measurements at a dilution of 10^–6^, 15 of 20 measurements at a dilution of 10^–7^ and 4 of 20 measurements at a dilution of 10^–8^. In contrast, after QIAamp extraction, RRT-PCR detected flu-v in all measurements at a dilution of 10^–6^, 14 of 20 measurements at a dilution of 10^–7^ and 2 of 20 measurements at a dilution of 10^–8^.

**Table 1:** Analytical sensitivity and precision comparisons of Maxwell 16-S and QIAamp method.

	Sensitivity comparison			Precision comparison (CV %)					
				Intra-run			Inter-run		
Extraction method	10^–6^	10^–7^	10^–8^	10^–2^	10^–4^	10^–6^	10^–2^	10^–4^	10^–6^
QIAamp	20/20^a^	14/20	2/20	1.05	0.62	1.42	0.81	0.42	2.09
Maxwell 16-S	20/20	15/20	4/20	1.44	1.19	1.46	1.07	1.62	1.57

a number of measurements with positive detection of flu virus RNA.

CV denotes coefficient of variation.

### Precision and Cross-contamination

The effect of automated sample preparation on the precision of RRT-PCR was estimated by extracting 10^–2^, 10^–4^ and 10^–6^ dilutions of the pH1N1SWL02 stock with the Maxwell 16-S compared to the QIAamp method. Quadruplicates per dilution for each of 4 runs were performed on 4 consecutive days. Both methods achieved high precision, i.e., the intra- and inter-run coefficients of variation (CV) ranged from 1.19% to 1.46% and 1.07% to 1.62% for the automated procedure, and from 0.62% to 1.42% and 0.42% to 2.09% for the manual method over the three measured concentrations (Table 1). To address the possibility of cross-contamination between samples within the Maxwell 16 Instrument, 3 batches of 8 reagent-blank samples were co-extracted in an alternating pattern with the high-titer virus samples used in precision determination across the cartridge rack. No false-positive results of RRT-PCR were observed in the blank samples.

### Detection of Flu-v in Various Samples

All respiratory samples after extraction by either the Maxwell 16-S or QIAamp method exhibited positive RP reactions by RRT-PCR with Ct values less than 37. The differences between the median Ct values of RP obtained with Maxwell 16-S and QIAamp Kit were –2.13 (*P*<0.001) for throat swabs and –0.95 (*P* = 0.001) for BALFs (Table 2). All throat swabs after extraction with the two methods also tested positive for flu A or B virus by RRT-PCR. The Maxwell 16-S yielded lower Ct values in 42/49 throat swabs than the QIAamp method, with the median Ct difference being –0.64 cycles (*P*<0.001) (Table 2). Of the 32 BALFs, the Flu A RRT-PCR detected 31 positives (96.9%) after Maxwell 16-S extraction and 29 positives (90.6%) after QIAamp extraction, and the only one missed after Maxwell 16-S extraction exhibited a weak positive reaction (Ct = 39.52) after QIAamp extraction. There was no statistical difference (*P* = 0.625) in the detection rates between the two methods. However, the Maxwell 16-S gave lower Ct values in 27/32 BALFs than the manual method, with the median Ct difference being –1.35 cycles (*P*<0.001) (Table 2). Furthermore, the Ct values of 31 mock-infected BALFs were delayed 0.76 cycles after Maxwell 16-S extraction and 1.74 cycles after QIAamp extraction, respectively, relative to the Ct values of controls containing the same amount of the virus (pH1N1SWL02).

**Table 2:** Comparison between Maxwell 16-S and QIAamp method for RNA extraction from throat swabs and BALFs^a^.

		Median Ct (IQR) (No. detected)		
Sample type (*n*)	Assay target	Maxwell 16-S	QIAamp	Ct difference
Throat swab (49)	Flu virus	26.67 (7.82) (49)	27.31 (7.50) (49)	–0.64*
	RNase P	27.29 (2.94) (49)	29.42 (3.59) (49)	–2.13*
BALF (32)	Flu virus	30.05 (0.87) (31)	31.40 (1.99) (29)	–1.35*
	RNase P	23.62 (2.89) (32)	24.57 (3.61) (32)	–0.95*

a Both the CDC Flu A/B Real-time RT-PCR and RP Real-time RT-PCR assays were performed after RNA extraction for detection of flu virus and human RNase P gene, respectively, from throat swabs and bronchoalveolar lavage fluids (BALFs). A Ct value of 45 was used to represent a negative sample result. IQR, interquartile range. Threshold cycle (Ct) difference is Maxwell 16-S minus QIAamp.

* *P*<0.05.

Analysis of fecal extracts by the Flu A RRT-PCR showed that the median Ct value differences between the Maxwell 16-S and QIAamp methods were –0.04 (*P* = 0.307) in poultry fecal pools and 1.40 (*P* = 0.234) in human fecal pools (Table 3). Their positive rates were 34/42 (81.0%) vs 39/42 (92.9%) in poultry fecal pools (*P* = 0.125), 12/18 (66.7%) vs 14/18 (77.8%) in human fecal pools (*P* = 0.688). There were no statistical differences in the Ct values and detection rates. Nevertheless, the Ct values of mock-infected poultry fecal samples (n = 32) and human fecal samples (n = 18) were increased by 1.55 and 4.72 cycles, respectively, for the Maxwell 16-S and 0.71 and 2.65 cycles, respectively, for the QIAamp method compared to those of viral controls. The results indicate that more RT-PCR inhibitors were co-extracted with viral RNA by Maxwell 16-S than by QIAamp Kit, which led to more false negatives. Of the 14 false-negative samples following Maxwell 16-S extraction, 9 turned positive after a 10-fold dilution of their extracts. To improve the capacity of the Maxwell 16 System to remove inhibitors from fecal matrix in a simple and effective way, Maxwell 16-M (100-µl input and 100-µl output) was adopted for further investigation. With Maxwell 16-M extraction, RRT-PCR detected flu-v in all fecal samples and gave improved Ct values for weak-positive samples found by Maxwell 16-S extraction (Appendix S1).

**Table 3:** Comparison of Maxwell 16-S, Maxwell 16-M and QIAamp method for extraction of influenza virus RNA from pooled fecal samples^a^.

	Median Ct (IQR) (No. detected)			Ct difference	
Feces origin (*n*)	Maxwell 16-S	Maxwell 16-M	QIAamp	Ct_S−Q_	Ct_M−S_
Poultry (42)	29.18 (4.94) (34)	30.36 (2.81) (42)	29.22 (2.25) (39)	–0.04	1.18
Human (18)	32.91 (15.47) (12)	30.69 (4.11) (18)	31.51 (9.00) (14)	1.40	–2.22*

a The CDC Flu A Real-time RT–PCR assay was performed after RNA extraction from fecal pools with the three extraction protocols. Since a known amount of virus was added to all the fecal samples except for ten naturally infected samples, no internal positive control was introduced into the PCR assay. Each pooled fecal sample (10%, W/V) consisted of feces from three individuals of the same type. A Ct value of 45 was used to represent a negative sample result. IQR, interquartile range. Ct_S**−**Q_ is Maxwell 16-S minus QIAamp. Ct_M**−**S_ is Maxwell 16-M minus Maxwell 16-S.

* *P*<0.05.

### User Convenience of the Maxwell 16 System

The estimated hands-on time was reduced by ca. 31 min for extraction of 6 samples and by ca. 39 min for 16 samples with the automated method compared to the manual method, although both methods were almost equivalent in total extraction time (Table 4). The list prices (cost per extraction) of the kits for Maxwell 16 System and QIAamp method are similar in China, but the former is much more expensive than the latter in the United States (Table 4). Still, the Maxwell 16 System features other conveniences: (i) any number of specimens (up to 16) can be processed per run; (ii) viral DNA and RNA can be co-purified, which was particularly useful for screen testing of multi-pathogens such as diverse respiratory viruses in one specimen [6]; iii) very little maintenance and training are required due to the prefilled reagent cartridge and simplified design.

**Table 4:** Comparison of time and cost between the manual and automated extraction method.

Extraction method	Cost/specimen^a^	No. of extracted samples	Total time (min)/run^b^	hands-on time (min)/run
Maxwell 16 System	$7.2, ¥65	6	67	17
		16	87	38
QIAamp	$4.4, ¥58	6	58	48
		16	87	77

a List prices in China (Chinese Yuan) and in the US (US dollar) for kits not including materials to be supplied by user; For QIAamp method, the list price was for the kit with reagents for 50 extractions.

b Timing began with addition of the lysis buffer and concluded with the recovery of RNA.

## Discussion

Earlier reports found that magnetic particle-based automated systems could be inferior [10], close [11], [12] or superior [4] to manual column methods for recovering viral RNA from various sample types, moreover, the automated systems generally brought about high precision and hands-on time reduction. Our data demonstrated that Maxwell 16-S RNA extraction had good linearity and precision over a wide concentration range and higher sensitivity in detection of stock flu-v in VTM than the QIAamp method.

To extend its applicability to clinical and field samples, the Maxwell 16-S was tested for RNA extraction from respiratory and fecal samples from humans as well as poultry. The performance of this automated procedure on the respiratory samples (including throat swabs and BALFs) was largely in agreement with its analytic performance on the virus stock. That is, greater sensitivity reflected by the lower Ct values for both the viral and cellular RNA was obtained from Maxwell 16-S compared with the QIAamp method. In contrast to the reference method and indicated by internal positive control, fewer or no PCR inhibitors existed in the throat swabs after Maxwell 16-S extraction. Similar findings were noted by Reznikov et al. [13], who found that throat swabs were free of PCR inhibitor, and by Loens et al. [4], who validated that a magnetic particle-based automated system extracted NA more efficiently from throat swabs (higher recovery and/or fewer inhibitors) than a QIAamp column method, and observed that PCR inhibitors existed in fewer throat swabs. BALFs contained high concentrations of compounds inhibiting PCR that could not be removed by various methods [14]. In our study, comparison of Ct values between viral controls and the virus-spiked BALFs extracted by the same method clearly revealed that these inhibitory effects of BALFs on RT-PCR were reduced after extraction by Maxwell 16-S compared to the QIAamp method, which might contribute to the higher sensitivity of Maxwell 16-S in this sample matrix.

Fecal material (feces, rectal or cloacal swabs) is an alternative matrix for detection of flu-v in poultry as well as in humans [15], [16], [17]. However, the fecal matrix has been recognized as a difficult and heterogeneous sample matrix for molecular analysis owing to its complex chemical composition and potential for PCR inhibition [3], [17], [18], [19], [20]. In our study, RNA extracts from fecal samples by Maxwell 16-S possessed more inhibitors than those obtained by the QIAamp method, which resulted in the reduced detection sensitivity of the former method. The inhibitors may act not only on the PCR amplification process, but also on pre-PCR processing procedures [21]. Therefore, a proper reduction in starting sample volume may outcompete dilution of extracts alone (also including an increase of elution volume) in removing inhibitors. This concept was supported by the findings that all the same fecal samples that showed false-negative results after Maxwell 16-S extraction and partially positive results when their extracts were diluted 10-fold were detected as positive after Maxwell 16-M extraction. The differential performances of Maxwell 16 System between the respiratory samples and fecal samples suggest that an increase in input and/or a decrease in output is beneficial for NA detection for some sample matrices with concentrated NA, but may be detrimental for other sample matrices that possesses increased inhibitors, and vice versa. Supporting evidence also comes from the detection of SARS coronavirus by RRT-PCR, the sensitivity of which increased with increased input of nasopharyngeal aspirate or human plasma [22], [23], whereas remained the same or decreased with increased input of feces [18]. Moreover, in our study, the Ct values of fecal samples after Maxwell 16-M extraction were improved in samples with higher inhibitor concentrations but delayed in samples with no or fewer inhibitors after Maxwell 16-S extraction (Appendix S1). Therefore, the sample volume should be optimized to balance the recovery of RNA and the removal of inhibitors in the application of the Maxwell 16 System to every sample type.

The risk of cross-contamination between samples is a concern due to the full opening of reagent cartridges during automated sample processing. However, no cross-contamination of negative samples by adjacent strongly positive samples occurred in our assays. The Maxwell 16 System utilizing magnetic particles to handle sample transfer and disposable plungers that shield magnetic handlers might both contribute to minimizing the possibility of cross-contamination.

In conclusion, the performance characteristics of the Maxwell 16 System enable its use for diagnosis of flu and viral load determination. However, this system possesses different abilities to remove inhibitors from respiratory samples and fecal samples, which subsequently exert an effect on the detection sensitivity. For challenging samples such as feces, a proper reduction in starting sample volume may improve the detection of flu-v. In spite of the initial investment requirement for the instrument and the relatively high cost of the kit, this system offers the distinct advantages of flexible sample throughput, co-extraction of viral RNA and DNA, reduction of hands-on time, minimal maintenance and training and little or no risk of cross-contamination.

## Supporting Information

Appendix S1 Detailed Ct values obtained for pooled fecal samples after extraction by Maxwell 16-S, Maxwell 16-M and QIAamp method*.
(XLS)
